# The Effects of Social Networks and Digital Technology on Non-suicidal Self-Injury in Children and Adolescents: A Systematic Review

**DOI:** 10.7759/cureus.101014

**Published:** 2026-01-07

**Authors:** David Galindo, Andres Lopez, Daniela Osorno, Lesmer Galindo Ruiz, Isabella Osorno

**Affiliations:** 1 Department of Psychiatry, Remington University Corporation, Medellin, COL; 2 Department of Child and Adolescent Psychiatry, Universidad El Bosque, Bogotá, COL; 3 Department of Medicine, Remington University Corporation, Medellin, COL

**Keywords:** children and adolescents, digital health interventions, effects of social media, non-suicidal self-injury, self-harm

## Abstract

Non-suicidal self-injury (NSSI) constitutes a significant and increasingly prevalent clinical problem among children and adolescents. At the same time, social media and digital technologies have become integral to adolescents’ social interaction, emotional regulation, and identity development, raising concerns about their potential contribution to NSSI risk as well as their role in prevention and intervention. This systematic review synthesizes current evidence on the relationship between digital platform use and NSSI in pediatric and adolescent populations.

Thirteen studies encompassing randomized controlled trials, observational designs, and qualitative research were included. Across study types, higher levels of social media use and digital engagement were consistently associated with an increased likelihood of NSSI, self-harm behaviors, depressive symptoms, and suicidal ideation, with effects more pronounced among female adolescents. Several population-based studies demonstrated dose-response relationships, particularly for social networking platforms, suggesting that intensity and modality of use are clinically relevant. Notably, the type and purpose of digital activity appeared more informative than total screen time alone, as structured or knowledge-oriented digital engagement showed neutral or potentially protective associations.

Evidence from randomized trials indicated that digitally delivered interventions targeting emotion regulation are feasible, safe, and associated with meaningful reductions in NSSI frequency, alongside improvements in psychosocial functioning and quality of life. Across study designs, emotional dysregulation emerged as a central mechanism linking digital experiences to self-injurious behavior.

In summary, digital environments play a complex and multifaceted role in adolescent NSSI, functioning both as potential risk contexts and as platforms for therapeutic intervention. These findings support the need for nuanced, developmentally informed clinical assessments of digital behavior and the integration of gender-sensitive, emotion-focused digital strategies into comprehensive prevention and early intervention approaches for adolescents at risk of NSSI.

## Introduction and background

Non-suicidal self-injury (NSSI) has been increasingly recognized as a clinically significant phenomenon within contemporary mental health research, particularly among adolescent and young adult populations [1]. NSSI refers to the intentional and direct destruction of one’s own body tissue in the absence of suicidal intent and encompasses behaviors such as cutting, burning, hitting, or scratching [2]. Although these acts are not driven by a desire to die, substantial empirical evidence indicates a strong association between NSSI and heightened psychological distress, psychiatric comorbidity, and an increased risk of subsequent suicidal ideation, suicide attempts, and suicide completion, underscoring its relevance as an important prognostic indicator [3].

From a psychopathological perspective, NSSI is conceptualized as a maladaptive coping mechanism predominantly aimed at modulating aversive emotional states, reducing internal tension, enacting self-punishment, or externally expressing psychological distress when adaptive regulatory strategies are insufficient [4]. Its onset and persistence are particularly common during adolescence, a developmental period characterized by ongoing neurobiological maturation, emotional lability, and increased sensitivity to interpersonal and contextual stressors [5]. The rising prevalence of NSSI in this population has positioned it as a major concern for clinical services and public health systems [6]. Notably, despite its clinical relevance, help-seeking remains limited: our results showed that only 15.64% of secondary school students who engaged in NSSI sought clinical consultation, highlighting a substantial treatment gap and the likelihood that a large proportion of affected adolescents remain undetected within healthcare systems [6].

Concurrently, the rapid expansion of digital technologies has profoundly altered patterns of social interaction, emotional expression, and information exchange. Online platforms now constitute a central context in which adolescents engage with peers, construct self-identity, and seek social validation [7]. These environments provide continuous exposure to peer-generated content and social feedback, which may influence emotional regulation processes and behavioral responses. While digital platforms can facilitate interpersonal connection and access to supportive resources, accumulating evidence suggests that certain patterns of engagement may be associated with increased psychological vulnerability.

Several theoretical and empirical frameworks have been proposed to explain how digital environments may interact with risk for NSSI. Recurrent exposure to highly curated and idealized representations of others may contribute to negative self-evaluation, affective dysregulation, and increased emotional distress. These mechanisms may be particularly salient during adolescence, when self-concept formation is ongoing and sensitivity to external evaluation is heightened [8]. In this context, digital interactions may amplify emotional strain and increase reliance on maladaptive coping strategies, including self-injurious behaviors.

Interpersonal stressors occurring within online settings represent an additional dimension of risk. Experiences involving online hostility, social exclusion, or repeated exposure to demeaning interactions may exacerbate feelings of rejection, shame, and psychological pain. The persistent, highly visible, and often uncontrollable nature of digital interactions may further intensify emotional dysregulation, thereby increasing susceptibility to NSSI among vulnerable individuals. Moreover, exposure to content depicting or discussing self-injury may contribute to behavioral reinforcement through processes of normalization, desensitization, or imitation.

Importantly, digital environments are not uniformly detrimental. Emerging evidence suggests that certain online platforms, moderated digital communities, and mobile health applications may exert protective effects by promoting mental health literacy, adaptive emotion regulation strategies, peer support, and timely help-seeking behaviors [9]. When appropriately designed and implemented, these digital interventions may contribute to resilience and symptom reduction, highlighting the need for a balanced and nuanced evaluation of digital influences on adolescent mental health [10].

Despite growing scientific interest, the relationship between digital platform use and NSSI remains incompletely understood. Existing studies are characterized by substantial methodological heterogeneity, variability in operational definitions, and inconsistent outcome measures, limiting the generalizability and clinical applicability of findings [11]. Furthermore, many healthcare professionals, particularly in primary care settings, lack standardized screening instruments and evidence-based guidance for the early identification and management of NSSI, underscoring a critical gap between research evidence and clinical practice [12].

In light of these limitations, the present systematic review aims to synthesize the available evidence regarding the association between digital platform use and NSSI among adolescents. Specifically, this review seeks to identify digital-related risk and protective factors and to inform the development of preventive, diagnostic, and early intervention strategies applicable in clinical and community contexts [13]. A clearer understanding of how digital environments interact with adolescent psychological vulnerability is essential for advancing evidence-based approaches to the prevention and management of NSSI [14].

## Review

The available evidence indicates that the relationship between social media use and adolescent mental health is complex, heterogeneous, and strongly context-dependent rather than uniform or linear. Although multiple studies report associations between greater exposure to digital environments and increased risk of NSSI, depressive symptoms, and emotional distress, these findings are not consistent across outcomes and cannot be explained solely by the amount of time spent online [15]. This variability reflects both methodological limitations and the inherent difficulty of capturing the diversity of digital experiences through a single metric.

Digital platforms have become structurally embedded in adolescents’ daily lives, functioning as spaces for social interaction, identity formation, and emotional regulation. Within this context, not all patterns of digital engagement exert the same psychological impact [16]. Evidence suggests that certain forms of online interaction may intensify emotional vulnerability, particularly when characterized by social comparison, external validation, or repeated exposure to emotionally charged content. Conversely, other forms of engagement may serve adaptive functions, especially for adolescents with limited offline social support [17].

Simplistic classifications of digital behavior are insufficient to explain its psychological effects [18]. Beyond observable levels of activity, clinically relevant factors include the purpose of use, the subjective meaning attributed to digital interactions, and their role within the adolescent’s emotional and interpersonal functioning. Conceptualizing digital media use as a homogeneous behavior reduces analytical precision and limits the interpretability of findings from both empirical and clinical perspectives.

Taken together, these considerations highlight the need to move beyond reductionist approaches and toward integrative models that account for the type of digital activity, emotion regulation processes, individual vulnerability, and broader psychosocial context. Such frameworks are essential for advancing a more precise understanding of how digital environments contribute to the development and maintenance of NSSI during adolescence [19]. 

Methods

Eligibility Criteria and Search Strategies

This systematic review was conducted in accordance with the Preferred Reporting Items for Systematic Reviews and Meta-Analyses (PRISMA) 2020 guidelines to ensure methodological rigor, transparency, and reproducibility throughout the research process. The Population, Intervention, Comparison, and Outcome (PICO) framework was used to define the search strategy and study selection criteria. The population consisted of children and adolescents aged 7-18 years. The intervention was defined as the use of social networks and digital technologies, including internet access, smartphones, and digital platforms. The comparison group included individuals with no use or lower levels of exposure to social networks and digital technologies. The outcome of interest was the presence of NSSI. 

To further ensure transparency and reproducibility, the protocol for this systematic review was prospectively registered in PROSPERO (registration number: CRD42024580002).

The literature search was restricted to peer-reviewed articles published in English and Spanish between January 2000 and August 2024. This restriction may introduce language bias; however, it was applied to ensure consistency in methodological assessment and data interpretation.

A comprehensive search was conducted across multiple electronic databases, including PubMed, MEDLINE, ScienceDirect, PsycINFO, ClinicalKey, ClinicalTrials.gov, and Google Scholar, selected due to their extensive coverage of biomedical, psychological, and clinical research. Relevant keywords and controlled vocabulary terms were combined using Boolean operators to enhance search sensitivity and specificity. The complete search strategy is presented in Table 1.

**Table 1:** Keywords used for the literature search

Category	Details
Databases searched	PubMed, MEDLINE, ScienceDirect, PsycINFO, ClinicalTrials.gov, Google Scholar
Time frame	January 2000 – August 2024
Language	English and Spanish
Search strategy	#1 AND #2 AND #3
#1 (Population)	"Children" OR "Adolescents" OR "Youth"
#2 (Exposure)	"Social networks" OR "Social media" OR "Digital technology" OR "Internet" OR "Smartphones" OR "Social networking platforms"
#3 (Outcome)	"Non-suicidal self-injury" OR "Self-harm" OR "Deliberate self-injury"

Inclusion Criteria

Studies were considered eligible for inclusion if they met predefined criteria. Eligible studies included participants aged 7-18 years, encompassing children and adolescents. The condition of interest was NSSI examined in relation to the use of social networks and digital technologies. Acceptable study designs comprised clinical trials, cohort studies, cross-sectional studies, and longitudinal studies. Only studies published between 2000 and 2024 were considered. Publications were required to be written in English or Spanish and to be peer-reviewed articles indexed in the selected electronic databases.

Exclusion Criteria

Studies were excluded if they met any of the following criteria: publication types such as theses, monographs, editorials, letters to the editor, book reviews, news articles, or opinion papers; inclusion of participants outside the defined age range of 7-18 years; focus on suicidal behavior or other forms of self-harm not specifically related to NSSI or not explicitly associated with social network use; or failure to meet acceptable methodological standards, including lack of adherence to PRISMA-based reporting principles.

Screening Process

All records identified through the literature search were imported into a reference management software to facilitate duplicate removal. Titles and abstracts were independently screened by two reviewers according to the predefined inclusion and exclusion criteria. Studies deemed potentially eligible underwent full-text assessment. Disagreements between reviewers were resolved through discussion, and when consensus could not be reached, a third reviewer was consulted for final adjudication. The study selection process was documented using a PRISMA flow diagram, detailing the number of records identified, screened, excluded, and included at each stage.

Data Extraction

Data extraction was carried out using a standardized extraction form developed a priori. The extracted variables included study characteristics such as authors, year of publication, country, and study design; participant characteristics including age range and gender distribution; exposure characteristics such as type of social network or digital platform and duration and frequency of use; and outcome measures including incidence or prevalence of NSSI, assessment instruments, and severity indices. Key findings related to the association between social network use and NSSI were systematically recorded. Data extraction was performed independently by two reviewers, with discrepancies resolved through consensus or, when necessary, consultation with a third reviewer. All extracted data were entered into a structured Excel database.

Risk of Bias and Quality Assessment

Methodological quality and risk of bias were assessed according to study design. Randomized and experimental studies were evaluated using the Cochrane Risk of Bias Tool version 2.0, whereas observational studies were appraised using the Newcastle-Ottawa Scale, with particular emphasis on participant selection, group comparability, and outcome assessment. Quality assessment was conducted independently by two reviewers, and discrepancies were resolved by consensus.

Data Synthesis

Given the anticipated methodological heterogeneity across studies, particularly in study design, exposure definitions, outcome measurements, and assessment instruments, a narrative synthesis approach was adopted. Findings were organized into thematic domains, including incidence and prevalence of NSSI among social network users; impact of social network and digital technology use on the onset or severity of NSSI; and moderating factors such as cyberbullying, social comparison, gender, age, and psychiatric vulnerability. Where sufficient data were available, subgroup analyses were conducted based on demographic characteristics, type of social networking platform, and duration or intensity of use. A quantitative meta-analysis was not conducted due to substantial heterogeneity among the included studies.

Results

A comprehensive and systematic search of electronic databases and registers, including PubMed, MEDLINE, PsycINFO, ScienceDirect, and Google Scholar, supplemented by additional sources, yielded a total of 183 records. Following the removal of duplicate records (n=24), 159 records remained. Of these, 85 records were excluded based on title and abstract screening as they were deemed ineligible according to the predefined inclusion criteria. Consequently, 74 records proceeded to formal title and abstract screening.

After independent screening of titles and abstracts, 18 records were excluded due to lack of relevance to the study objectives. Subsequently, 56 reports were sought for full-text retrieval; however, 28 reports could not be retrieved despite reasonable efforts. The remaining 28 full-text articles were assessed for eligibility.

Upon full-text evaluation, 15 studies were excluded for predefined methodological and content-related reasons, including inappropriate outcome measures (wrong outcome; n=7), non-relevant interventions (wrong intervention; n=5), and publication in a non-eligible language (n=3). Ultimately, 13 studies fulfilled all eligibility criteria and were included in the final qualitative synthesis of this systematic review.

**Figure 1:**
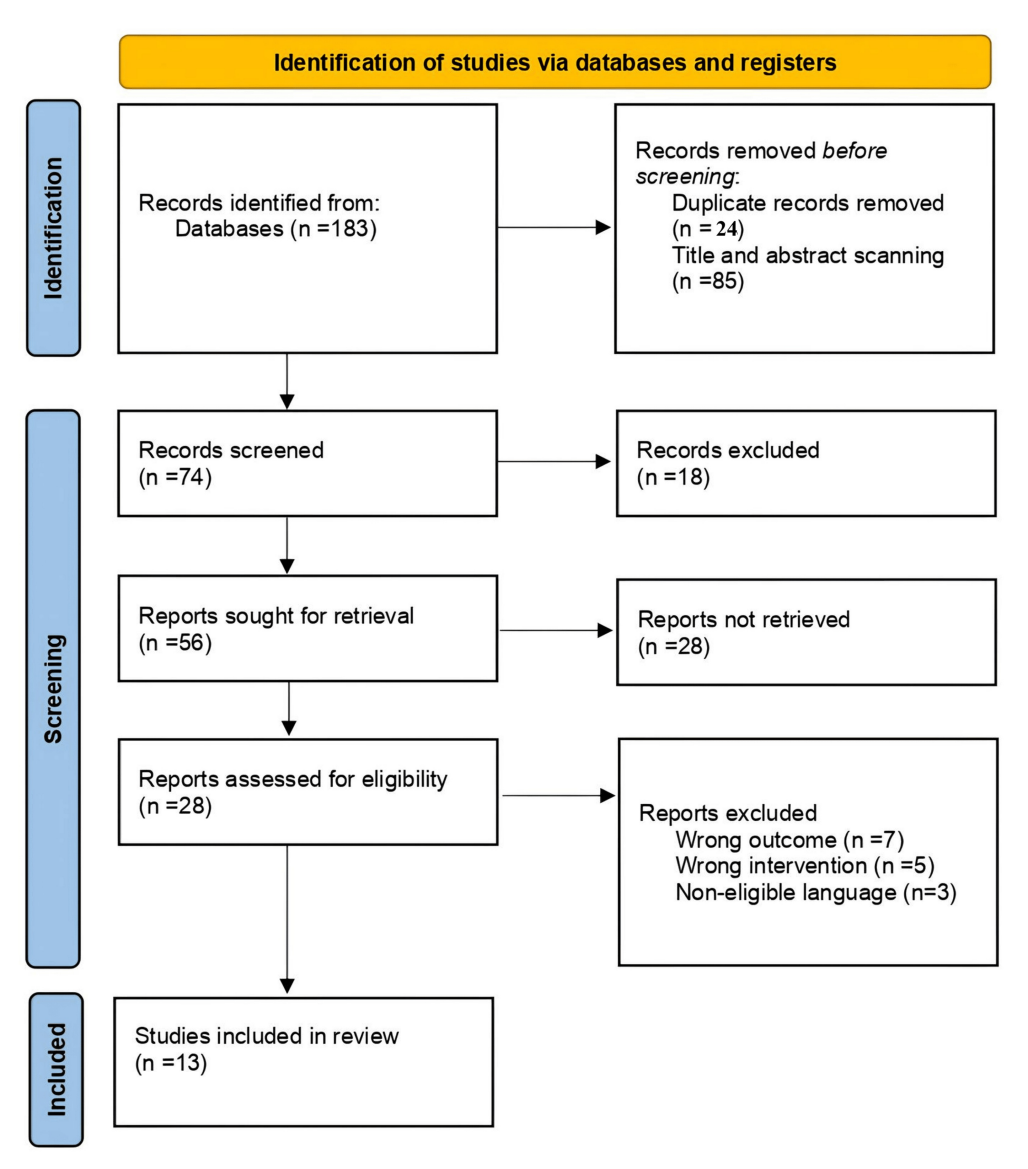
PRISMA flow diagram indicating the study selection and inclusion process PRISMA: Preferred Reporting Items for Systematic Reviews and Meta-Analyses

Figure 1 presents the PRISMA flow diagram illustrating the study identification, screening, eligibility assessment, and final inclusion process.

Table 2 presents the main characteristics of the 13 studies included in the systematic review, detailing study design, population demographics, and outcomes related to NSSI. Collectively, these studies examine pediatric and adolescent populations and report associations between exposure to social networks and digital technologies, NSSI-related behaviors, and the impact of targeted interventions on self-harm severity and psychosocial functioning.

**Table 2:** Summary of included studies on social network use and non-suicidal self-injury in children and adolescents This table summarizes the main characteristics of the studies included in the systematic review, including authorship, study design, population characteristics, and outcomes related to non-suicidal self-injury and exposure to social networks and digital technologies. CAMHS: Child and adolescent mental health care; NSSI: non-suicidal self-injury

Authors	Study design	Population	Outcomes relevant to the systematic review
Pontén et al. (2024) [20]	Randomized controlled trial	Adolescents aged 13–17 years with NSSID	Reduction or absence of NSSI at one month post intervention; predictive accuracy of clinical judgment versus machine learning models for short-term NSSI outcomes
Gámez-Guadix et al. (2020) [21]	Mixed methods study	Adolescents aged 12–18 years	Prevalence and motivations of online self-injury; role of digital environments in initiation and maintenance of NSSI
Bjureberg et al. (2023) [22]	Multicenter superiority randomized controlled trial	Adolescents aged 13–17 years with NSSID	Weekly frequency of NSSI; improvements in emotion regulation and global functioning
Morrish et al. (2024) [23]	Randomized controlled trial with cost-effectiveness analysis	Adolescents aged 12–17 years receiving CAMHS	Changes in self-harm severity, health-related quality of life, and healthcare utilization associated with the intervention
Stallard et al. (2024) [24]	Single blind randomized controlled trial	Adolescents aged 12–17 years receiving CAMHS	Reduction in self-harm frequency and severity; changes in depressive and anxiety symptoms
Shafi et al. (2021) [25]	Retrospective case-control study	Adolescents hospitalized in a psychiatric unit	Association between social media use and presence of NSSI, severity of suicidal ideation, and suicide risk at admission
McAllister et al. (2021) [26]	Cross-sectional cohort study (MCS)	Adolescents aged 13–15 years	Association between time spent on digital media, self-harm behaviors, and depressive symptoms
Yang et al. (2020) [27]	Cross-sectional study	Adolescents and young adults aged 12–24 years	Association between frequency of internet use and NSSI, suicidal ideation, and gender specific risk patterns
Brown et al. (2020) [28]	Qualitative study	Adolescents aged 16 years or older	Perceived impact of exposure to NSSI-related content on emotional state and self-injurious behavior
Barthorpe et al. (2020) [29]	Cross-sectional cohort study (MCS)	Adolescents aged 13–15 years	Dose response relationship between social media use, self-harm, depressive symptoms, and self-esteem
Olsen et al. (2021) [30]	Feasibility randomized controlled trial	Adolescents aged 13–17 years with recurrent NSSI	Change in frequency of NSSI episodes post intervention; feasibility of a digital structured intervention targeting NSSI
Robertson et al. (2022) [31]	Cross-sectional study (ABCD cohort)	Children aged 9–10 years	Association between screen time, self-harm or NSSI, suicidal ideation, and internalizing disorders
Vente et al. (2020) [32]	Descriptive cross-sectional study	Adolescents aged 12–21 years	Co-occurrence of NSSI, intensity of social media use, and online risk behaviors such as sexting

Table 3 summarizes the main results and conclusions of the studies included in the systematic review, emphasizing clinical, behavioral, and psychosocial outcomes associated with NSSI and exposure to social networks and digital technologies. Overall, the findings indicate that digital media use is associated with increased vulnerability to NSSI, particularly among adolescents, with consistent evidence of gender-specific effects and the influence of key moderating factors such as emotional dysregulation, cyberbullying, and intensity of digital media use. Interventional studies suggest that digital and emotion regulation-focused interventions may reduce the frequency and severity of NSSI, although evidence regarding their superiority over usual care remains inconclusive. Collectively, these findings highlight the importance of integrating digital behavior assessment and emotion regulation strategies into preventive and clinical approaches for adolescents at risk of NSSI. 

**Table 3:** Summary of results and conclusions of studies on non-suicidal self-injury and digital media use NSSI: Non-suicidal self-injury

Authors	Results	Conclusions
Pontén et al. [20]	Clinical judgment predicted NSSI abstinence with 63 percent accuracy, while the machine learning model achieved 67 percent; both outperformed the baseline model at 49 percent. Emotional dysregulation was the strongest predictor in the machine learning model.	Clinical judgment and machine learning demonstrated comparable predictive performance. Difficulties in emotion regulation appear central to short term NSSI outcomes, supporting their relevance as a key therapeutic target.
Gámez Guádix et al. [21]	Online self-injury was reported by 18.6 percent of adolescents, with prevalence increasing with age. Motivations included emotional expression, help seeking, and imitation.	Online self-injury represents a prevalent and multifaceted phenomenon, combining emotional distress and social reinforcement, and warrants preventive and clinical attention in digital contexts.
Bjureberg et al. [22]	IERITA plus usual care resulted in significantly greater reductions in NSSI frequency compared with usual care alone, with effects maintained for up to three months. Improvements in emotion regulation mediated NSSI reduction.	IERITA is an effective intervention for adolescents with NSSID, with sustained benefits supported by improvements in emotion regulation.
Morrish et al. [23]	Both intervention and control groups showed reductions in self-harm. The BlueIce group demonstrated slightly greater improvement and lower healthcare costs, although differences were not statistically significant.	BlueIce appears feasible, low cost, and potentially cost effective as an adjunct to usual care, warranting larger trials with longer follow up periods.
Stallard et al. [24]	Significant improvements in self-harm and emotional symptoms were observed in both groups, with no statistically significant between group differences. Trends favored fewer hospital admissions and adverse events in the BlueIce group.	BlueIce is a safe adjunctive intervention that may reduce risk related outcomes, although its additional efficacy over usual care remains inconclusive.
Shafi et al. [25]	Social media use was associated with higher odds of NSSI at psychiatric admission, with an adjusted odds ratio of 2.55. Associations with suicidality were not statistically significant.	Social media use may function as a clinical marker of increased vulnerability to NSSI in hospitalized adolescents, supporting routine assessment during psychiatric evaluation.
McAllister et al. [26]	Associations between social media use, depressive symptoms, and self-harm were observed exclusively in girls, particularly with exposure exceeding two hours per day.	The mental health impact of digital media appears gender specific, with adolescent girls representing a higher risk subgroup for social media related harm.
Yang et al. [27]	Frequent social media use predicted higher odds of NSSI and suicidal ideation in both genders. Knowledge based platforms showed a protective effect among males.	The type of online activity, rather than overall screen time, is critical in understanding NSSI risk, underscoring the need for gender sensitive interventions.
Brown et al. [28]	Posting NSSI related images was motivated by the need for social connection and validation. More severe images received greater attention, potentially reinforcing self-injurious behavior.	Social media may provide temporary emotional support but can also reinforce NSSI through social validation, highlighting the need for safer online environments and interventions.
Barthorpe et al. [29]	In girls, each 30-minute increase in social media use was associated with higher risk of self-harm, depressive symptoms, and lower self-esteem. No significant associations were observed in boys.	Social media intensity may serve as a gender specific indicator of mental health vulnerability in adolescent females.
Olsen et al. [30]	No clinical outcomes were reported, as the study presented a predefined statistical analysis plan for a feasibility randomized controlled trial.	Predefined analysis plans enhance transparency, reduce bias, and improve reproducibility, providing a rigorous framework for future ERITA trials in adolescents with NSSI.
Robertson et al. [31]	Screen time equal to or greater than two hours per day was associated with increased risk of depression, self-harm, and suicidality. Dose response effects were strongest for digital media use.	High digital media exposure may function as a risk marker for internalizing disorders in late childhood, emphasizing the need for longitudinal research.
Vente et al. [32]	Use of four or more social media applications was associated with higher risk of self-harm and sexting. Bullying exposure and female sex were linked to greater self-harm prevalence.	Intensive and diversified social media use may increase engagement in high-risk behaviors, supporting the importance of targeted digital screening in adolescents.

Table 4 presents the risk of bias assessment of the included studies, conducted using design-specific methodological appraisal tools. Randomized controlled trials were evaluated with the Cochrane Risk of Bias Tool version 2 (RoB 2), observational studies were assessed using ROBINS-I, and qualitative studies were appraised with the Critical Appraisal Skills Programme (CASP) checklist. Risk of bias was classified as low, some concerns, moderate, or high according to the standardized criteria of each assessment instrument.

**Table 4:** Risk of bias assessment of included studies This table presents the risk of bias evaluation of the included studies, indicating study design, level of bias, assessment tool applied, and the rationale for each judgment. Risk of bias was assessed using RoB 2 for randomized trials, ROBINS-I for observational studies, and CASP for qualitative research.

Authors	Study design	Risk of bias	Tool used	Justification
Pontén et al. (2024) [20]	Randomized controlled trial	Low	RoB 2 (Cochrane)	Adequate randomization, low attrition, and blinded outcome assessment
Gámez-Guadix et al. (2020) [21]	Mixed-methods study	Moderate	ROBINS-I + CASP	Quantitative component cross-sectional; qualitative analysis methodologically robust
Bjureberg et al. (2023) [22]	Multicenter superiority randomized controlled trial	Low	RoB 2 (Cochrane)	Multicenter design, standardized measures, and low proportion of missing data
Morrish et al. (2024) [23]	Randomized controlled trial with cost-effectiveness analysis	Low	RoB 2 (Cochrane)	Prespecified outcomes, robust randomization, and validated instruments
Stallard et al. (2024) [24]	Single-blind randomized controlled trial	Some concerns	RoB 2 (Cochrane)	Participants not blinded; reliance on self-reported self-harm outcomes
Shafi et al. (2021) [25]	Retrospective case–control study	Moderate	ROBINS-I	Retrospective design, selection bias, and residual confounding
McAllister et al. (2021) [26]	Cross-sectional cohort study	Moderate	ROBINS-I	Potential confounding and self-reported digital exposure
Yang et al. (2020) [27]	Cross-sectional study	Moderate	ROBINS-I	Temporal ambiguity, recall bias, and limited control of confounders
Brown et al. (2020) [28]	Qualitative study	Low	CASP (Qualitative checklist)	Clear methodology, reflexivity addressed, and data saturation achieved
Barthorpe et al. (2020) [29]	Cross-sectional cohort study	Moderate	ROBINS-I	Dose–response relationship reported; causality cannot be inferred
Olsen et al. (2021) [30]	Feasibility randomized controlled trial	Some concerns	RoB 2 (Cochrane)	Small sample size, feasibility focus, and limited statistical power
Robertson et al. (2022) [31]	Cross-sectional cohort study	Moderate	ROBINS-I	Cross-sectional design, self-reported exposure and outcomes
Vente et al. (2020) [32]	Descriptive cross-sectional study	Moderate–High	ROBINS-I	Absence of a comparator group, descriptive design, and limited adjustment

Study Findings

This systematic review synthesized evidence from 13 studies examining the association between social media and digital technology use and NSSI in children and adolescents. The included literature comprised randomized controlled trials, observational studies, qualitative research, and mixed methods designs. Overall, the evidence demonstrates a consistent association between digital media exposure and NSSI-related outcomes, with variability according to study design, population characteristics, and the specific type of digital activity assessed.

Evidence from randomized and feasibility trials indicates that digitally supported interventions targeting adolescents with NSSI are associated with reductions in the frequency and severity of self-injurious behaviors, as well as improvements in emotion regulation, global functioning, and health-related quality of life [21]. ERITA-based interventions showed significantly greater reductions in NSSI compared with usual care, with effects mediated by improvements in emotional regulation and maintained over follow-up periods [22]. Interventions such as BlueIce demonstrated feasibility, safety, and potential cost effectiveness as adjuncts to standard care, although statistically significant differences between groups were not consistently observed [23,24]. The use of predefined statistical analysis plans in feasibility trials enhanced methodological transparency and reproducibility [25,30].

Predictive modeling studies showed that clinical judgment and machine learning approaches achieved comparable accuracy in predicting short-term NSSI outcomes, both outperforming baseline predictive models. Emotional dysregulation emerged as the strongest predictor, while clinician confidence and professional experience did not significantly improve predictive performance [20,26].

Observational- and population-based studies consistently reported that higher levels of social media use, screen time, or internet engagement were associated with increased likelihood of NSSI, self-harm behaviors, depressive symptoms, and suicidal ideation, particularly among female adolescents [27,29]. Several studies identified dose response relationships, with risk increasing beyond approximately two hours of daily exposure, and stronger associations observed for social networking platforms compared with other forms of digital media [31,33]. Intensive and diversified social media use, including engagement with multiple platforms, was associated with increased self-harm risk and online risk behaviors, particularly in the context of bullying exposure [32]. Importantly, engagement with knowledge-based digital platforms was associated with lower NSSI risk in male adolescents, suggesting that the type and purpose of digital activity may be more clinically relevant than overall screen time [27,34].

In clinical samples, adolescents hospitalized for psychiatric conditions who reported social media use demonstrated higher odds of presenting with NSSI at admission, supporting the interpretation that patterns of digital engagement may function as clinical markers of increased vulnerability, rather than isolated causal factors [25,34].

Qualitative and mixed methods studies highlighted the dual role of social media in NSSI. Digital platforms were described as spaces for emotional expression, validation, and social connection, but also as environments that may reinforce self-injurious behaviors through repeated exposure to NSSI-related content and social validation mechanisms [21,28].

In summary, digital and social media environments play a multifactorial role in the initiation, maintenance, and potential mitigation of NSSI in adolescents. While excessive or unregulated exposure, particularly to social networking content, appears to increase vulnerability [25,26], structured digital interventions and specific forms of online engagement may exert protective effects, especially when focused on improving emotional regulation [22,24]. These findings emphasize the need for developmentally informed and gender sensitive assessments of digital behavior, integrating emotion regulation processes into comprehensive prevention and intervention strategies for adolescents at risk of NSSI.

Discussion

The present systematic review integrates evidence from 13 studies, including randomized controlled trials, observational investigations, and qualitative research, to clarify the relationship between social media and digital technology use and NSSI in children and adolescents [20]. Overall, the findings indicate a consistent association between specific patterns of digital engagement and NSSI-related outcomes, although the magnitude and direction of these associations varied according to study design, population characteristics, and the type of digital activity assessed [21].

Evidence derived from randomized controlled trials suggests that digitally supported interventions for adolescents with NSSI are feasible, safe, and associated with clinically meaningful improvements, particularly in emotion regulation, global functioning, and health-related quality of life [22]. Although some trials did not demonstrate statistically significant between-group differences in primary outcomes [23], favorable trends were consistently observed, including reductions in healthcare utilization, emergency service contacts, and adverse events [24]. Importantly, superiority trials demonstrated significant and sustained reductions in NSSI frequency when structured digital interventions were added to usual care, with improvements in emotion regulation mediating treatment effects [22]. These findings support transdiagnostic models conceptualizing NSSI as a maladaptive strategy for affect regulation rather than a purely impulsive behavior.

Predictive modeling evidence further reinforces the central role of emotional dysregulation in NSSI. Comparable accuracy was observed between clinical judgment and machine-learning approaches in predicting short-term NSSI outcomes, with difficulties in emotion regulation emerging as the most influential predictor [20]. The lack of improvement in predictive accuracy associated with clinician confidence or professional experience highlights the limitations of subjective risk estimation and underscores the potential value of objective, data-informed tools as adjuncts to clinical decision-making.

Observational and population-based studies consistently reported associations between higher levels of social media use, screen time, or internet engagement and increased likelihood of NSSI, self-harm, depressive symptoms, and suicidal ideation, particularly among female adolescents [25]. Large cohort studies demonstrated dose-response relationships, with risk increasing beyond approximately two hours of daily exposure and stronger associations observed for social networking platforms compared with other forms of digital media [26]. Notably, not all forms of digital engagement conferred equivalent risk; engagement with knowledge-based platforms was associated with a lower risk of NSSI in male adolescents, suggesting that the type and purpose of online activity may be more clinically relevant than total screen time alone [27].

Findings from clinical samples indicate that social media use may function as a marker of heightened vulnerability rather than an isolated causal factor. Adolescents hospitalized for psychiatric reasons who had documented social media use showed significantly higher odds of presenting with NSSI at admission [25]. These findings support the integration of structured assessments of digital behavior into routine adolescent psychiatric evaluations, particularly in high-risk clinical settings.

Qualitative and mixed-methods studies provided critical contextualization of quantitative findings by elucidating adolescents’ subjective experiences in digital environments [28]. Social media platforms were frequently described as spaces for emotional expression, validation, and social connection, especially among adolescents experiencing isolation or psychological distress. However, exposure to NSSI-related content, particularly when more severe material received greater visibility, was reported to trigger distress, reinforce self-injurious urges, and contribute to social contagion processes [28]. Similarly, online self-injury behaviors reflected complex motivations involving emotional regulation, help-seeking, and imitation, highlighting the role of digital environments in both the initiation and maintenance of NSSI [21].

Importantly, the reviewed evidence also indicates potential protective and therapeutic roles of digital technologies when appropriately designed and implemented. Digitally delivered interventions demonstrated acceptability, low implementation costs, and signals of clinical benefit, particularly as adjuncts to standard care [23]. These findings suggest that digital platforms should not be conceptualized solely as risk environments but also as potential vehicles for early intervention, psychoeducation, and crisis support, provided that safeguards against harmful content and maladaptive social reinforcement are adequately implemented [34].

Several limitations must be acknowledged. Most observational studies relied on cross-sectional designs and self-reported measures of digital media use, limiting causal inference and introducing recall bias [29]. Additionally, the majority of studies were conducted in high-income Western countries, potentially constraining generalizability. Gender differences were inconsistently examined, and male adolescents were underrepresented in clinical intervention trials [22]. These limitations underscore the need for longitudinal, developmentally informed research incorporating objective measures of digital engagement and culturally diverse samples.

In summary, this review demonstrates that social media and digital technologies play a multifaceted role in the risk and management of NSSI among adolescents. Excessive or unmoderated exposure particularly to social networking content appears to increase vulnerability to self-injury, especially among female adolescents, whereas targeted digital interventions and certain forms of online engagement may confer protective effects [33]. These findings support the need for nuanced, individualized assessments of digital behavior and for integrating gender-sensitive, emotion-focused strategies into comprehensive prevention and intervention frameworks for adolescents at risk of NSSI.

## Conclusions

This systematic review demonstrates that social media and digital technology use are meaningfully associated with NSSI in children and adolescents, with risk varying according to the intensity, type, and emotional context of digital engagement. Higher exposure to social networking platforms is consistently linked to increased NSSI, depressive symptoms, and suicidal ideation, particularly among female adolescents, whereas not all digital activities confer equivalent risk. Across study designs, emotional dysregulation emerged as a central mechanism underlying NSSI, and patterns of digital behavior appear to function as markers of vulnerability rather than isolated causal factors. Importantly, evidence from randomized trials indicates that structured, emotion-focused digital interventions are feasible and may reduce NSSI frequency and improve functioning when used as adjuncts to usual care. Taken together, these findings support a nuanced, developmentally informed approach to digital media assessment in adolescent mental health, emphasizing the need to move beyond screen-time metrics toward individualized evaluation of digital behaviors to inform prevention and early intervention strategies for youth at risk of NSSI.
